# The Assessment of Mercury Concentrations in Two Species of Edible Forest Mushrooms, *Aureoboletus projectellus* and *Imleria badia*, and Their Impact on Consumers’ Health

**DOI:** 10.3390/foods14040631

**Published:** 2025-02-13

**Authors:** Michał Skibniewski, Bartosz Skibniewski, Iwona Lasocka, Ewa Skibniewska

**Affiliations:** 1 Department of Morphological Sciences, Institute of Veterinary Medicine, Warsaw University of Life Sciences, Nowoursynowska Street 159, 02-776 Warsaw, Poland; 2 One Health Section, The Scientific Society of Veterinary Medicine Students, Faculty of Veterinary Medicine, Warsaw University of Life Sciences, Nowoursynowska Street 159, 02-776 Warsaw, Poland; bartosz_skibniewski@sggw.edu.pl; 3 Department of Biology of Animal Environment, Institute of Animal Sciences, Warsaw University of Life Sciences, Ciszewskiego Street 8, 02-786 Warsaw, Poland; iwona_lasocka@sggw.edu.pl

**Keywords:** Hg, American boletus, brown boletus, food safety

## Abstract

In recent years, the consumption of wild mushrooms in Central Europe has significantly increased. These mushrooms are increasingly recognized as a nutritious, low-calorie, and environmentally friendly food option. They are a valuable source of protein and are rich in vitamins and minerals; however, they can also accumulate toxic elements that may pose risks to human health. This study examined the mercury concentrations in the fruiting bodies of two edible forest mushroom species: *Aureoboletus projectellus* and *Imleria badia*. This study took into account the distribution of Hg in the two morphological parts of mushroom fruiting bodies—the caps and the stipes. The total mercury content of the mushroom samples was analyzed using an AMA-254 analyzer. Both mushroom species exhibited higher mercury concentrations in their caps than in their stipes, with levels measuring 0.048 mg·kg^−1^ dry matter (DM) for *Aureoboletus projectellus* and 0.055 mg·kg^−1^ DM for *Imleria badia*. The mercury content in the stipes was 0.032 mg·kg^−1^ DM for *Aureoboletus projectellus* and 0.025 mg·kg^−1^ DM for *Imleria badia*. The results obtained indicate that these species do not pose a health risk to consumers in terms of Hg content and can be a valuable addition to the human diet. They are also an indicator of the quality of the forest environment of the central coast of Poland, which should be considered free of mercury pollution.

## 1. Introduction

In Central Europe, including Poland, wild mushrooms are a popular culinary product known for their unique flavors. Mushroom picking has become a cherished tradition and a favorite pastime for many people. Traditionally, mushroom picking is a recreational activity enjoyed by millions of Polish families. Additionally, wild mushrooms are often sold by the roadside, at local markets, and in large retail chains in a processed form, mainly being dried or pickled [1,2]. While most of the mushrooms collected are for personal use, some are sold to processing plants for industrial processing and export. Data from the Central Statistical Office of Poland indicate that the number of purchased mushrooms has increased recently from 2599 tons in 2015 to 3503 tons in 2022 [3]. It should be noted here that these values depend on the season’s weather conditions, as in dry years, the supply of mushrooms is lower than in wet years. An additional factor to be considered is that most mushrooms are harvested for personal use and their consumption remains outside of official control, preventing normative statistical data from being drawn. It is estimated that currently, in Poland, around 200 species of mushrooms harvested from natural sites are used for food and medicinal purposes. The average consumption of forest mushrooms in Poland is a few kilograms per inhabitant per year. Still, among the population living in large, forested areas, it can reach about 35 kg per person throughout the year. Therefore, official government sources publish data on mushrooms in terms of their nutritional value and how to harvest and identify species [4]. In recent years, a new species of mushroom, known as the American boletus (*Aureoboletus projectellus*) or the slender golden bolete, has emerged in Poland. This North American species arrived in Europe at the beginning of the 21st century and was first described in the early 2000s [5,6]. Over the past decade, its range has expanded significantly. Mass occurrences of the American boletus can be found in Latvia, Lithuania, and northern Poland. Originally, *Aureoboletus projectellus* was only present along the Baltic Sea coast, but it has since begun to spread into new regions and has recently been discovered inland. The species is considered to be potentially invasive. *Aureoboletus projectellus* forms mycorrhizal associations with native European trees, such as the Scots pine (*Pinus sylvestris*), as well as with introduced species like the mugo pine (*Pinus mugo*) [5,6,7]. This mushroom typically grows in groups and is primarily found on sandy substrates in dry coniferous forests and in coastal and inland wooded marshes and heaths [7]. Since this species is still dynamically spreading along Polish forests, the mass appearance of this fungus makes it the dominant species harvested by mushroom pickers in some areas of the country. It is notable among native mushrooms in the bolete family for its distinctly furrowed and grooved stem surface and its cap which extends significantly beyond its edge. Due to its appealing taste and aroma, this mushroom is highly sought after by foragers.

Another native edible mushroom species widespread in Poland is the brown boletus (*Imleria badia*). This mushroom often grows in large groups, sometimes consisting of a dozen or even several dozen individuals. The brown boletus is typically found on sandy soils, thriving in deciduous and coniferous forests, particularly among oaks, pines, and birches. It forms a strong mycorrhizal association with pine trees. The brown boletus is easily recognizable due to its distinctive dull, matt-brown cap, which reaches up to 15 cm in diameter. Its hymenophore appears as yellow tubes. The mushroom’s stipe is light brown, often thin, and sometimes curved, though it can also be thickened. These mushrooms typically appear in late summer and autumn and are highly valued by mushroom foragers due to their flavor [2].

Mushrooms offer numerous health benefits due to their unique chemical and nutritional properties. They serve as a valuable dietary supplement and are recognized as excellent prebiotics. Additionally, mushrooms are a source of natural bioactive compounds, including bioavailable amino acids, vitamins, minerals, and trace elements. They contain elements essential for human health, such as phosphorus, potassium, sodium, calcium, magnesium, manganese, zinc, and copper. Furthermore, they provide a range of vitamins, primarily from the B group, including significant amounts of vitamin B_2_, niacin (B_3_), and folacin (B_9_), along with smaller amounts of vitamins B_1_, B_12_, C, D, and E [1,8,9,10,11].

Many mushroom species contain biologically active substances that have potential applications in preventing and treating serious diseases, including cancer and heart disease. For example, mushrooms can help to lower cholesterol and reduce the risk of hypertension [8,9,12,13,14,15,16,17,18,19,20,21]. The chitosans and polyphenols found in mushrooms have also demonstrated various beneficial effects, such as immunostimulation, antidiabetic properties, and antibacterial, antiviral, and antioxidant activities [7,8,22,23,24].

Mushrooms have a high water content, resulting in low energy values. They are also a source of dietary fiber, which is mainly insoluble, and contain polyunsaturated fatty acids [10,25,26,27,28].

Although forest mushrooms contain metallic elements and metalloids beneficial to health, they also accumulate potentially toxic trace elements [29,30,31,32,33,34,35,36,37,38]. One is mercury, categorized as a global pollutant affecting the environment, including humans and animals [39,40]. In Poland, this metal’s primary source of emissions is coal-fired power generation. It is estimated that it is responsible for about 71% of mercury emissions. In addition, there is the local impact of so-called low emissions caused by using coal as the primary energy source for heating homes [41]. Mercury has no physiological function in the body. Safe concentrations of this metal are difficult to determine due to the variety and toxicity of its forms of occurrence. The harmfulness of mercury depends, among other things, on the chemical form, the route of exposure, and the amount and duration of exposure. It remains in the environment for a long time and undergoes biomagnification, further increasing its danger. Issue arise from long-term exposure to small amounts of mercury, which can bioaccumulate over time. Acute poisoning from this metal is very rare [42,43,44,45,46]. Mercury, a typical heavy metal, selectively binds to the sulfhydryl groups in the proteins that comprise cellular structures. Since nearly all proteins contain these sulfhydryl groups that can react with mercury, exposure to this element can disrupt enzymatic reactions and damage the cellular structures formed by these proteins. Mercury compounds interfere with almost all enzyme reactions, including those that play a role in protein biosynthesis. This disruption can cause pathological changes in the nervous system, especially in the brain. Furthermore, mercury exposure can lead to various metabolic disorders, such as issues related to the kidneys, immune system, heart, motor function, fertility, and genetics. In severe cases, mercury exposure can even result in death [46,47,48]. Due to the potential for mercury to accumulate in the environment, including wild mushrooms, the consumption of mushrooms can pose a real health risk to consumers, especially children, the elderly, and people with chronic illnesses [44,46,47,48,49].

This study aimed to assess the mercury content in the caps and stipes of two commonly harvested edible mushroom species: the American boletus and the brown boletus. The assessment was based on the maturity of the fruiting bodies, comparing young and mature specimens, and it also evaluated the health risks mushroom consumers face.

## 2. Materials and Methods

**Figure 1:**
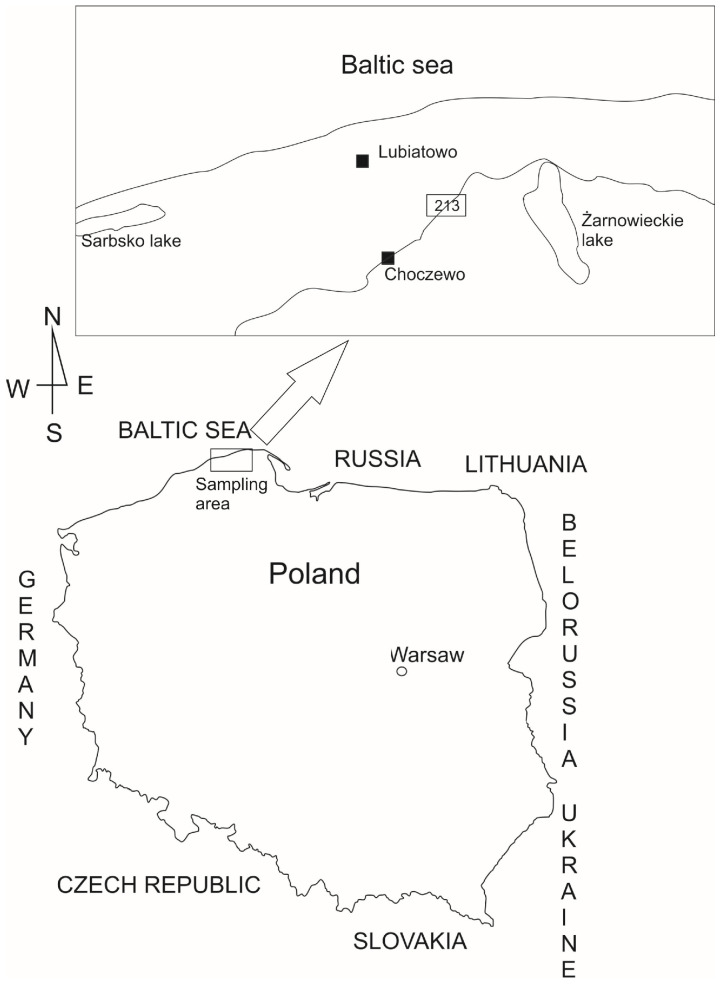
Schematic map of the sampling area and the 213-road.

Mushrooms were collected in late August and early September 2018 in Poland, specifically in the Pomeranian Voivodeship, within the Choczewo Forest District, and around the village of Lubiatowo. This area, located along the central Polish coast, is known for its unpolluted environment. The study sampling site is shown in Figure 1.

The collection site consisted of a forested region primarily featuring a mixed coniferous stand, dominated by Scots pine (*Pinus sylvestris*), with some bearded birch (*Betula pendula*) present as an admixture. Certain parts of the forest also included the introduced mountain pine (*Pinus mugo*). This habitat is typical for the mushroom species harvested in this study, including the American bolete (*Aureoboletus projectellus*) and the brown bolete (*Imleria badia*). Both species were identified using a taxonomic key [50]. The mushrooms collected on-site were cleaned of forest litter and other visible debris with a ceramic knife. A total of 194 mushroom fruiting bodies were collected, comprising 102 American boletes and 92 brown boletes. These mushrooms were then separated into caps and stems and measured using an electronic caliper. The detailed parameters of the harvested mushrooms are presented in Table 1.

The test material was stored frozen at −20 °C until analysis. After thawing, the caps and stems were separated, then cut into thin slices and dried (40 °C for ~24 h) in a laboratory oven with forced circulation (Memmert GmbH & Co. KG, Schwabach, Germany). Then, the caps and stems were ground separately in the laboratory grinder (A11 Basic, IKA, Warsaw, Poland) to obtain a homogeneous sample. For the analysis, approximately 50 mg of the mushroom cap and 50 mg of the stem were weighed using an analytical balance (Radwag E425, Radom, Poland). The total mercury content was determined using an atomic absorption spectrometer (Altec, Prague, Czech Republic). The samples prepared for analysis were placed directly in the nickel nacelle of the analyzer and automatically introduced into the furnace chamber, as this device did not require prior digestion of samples. This procedure was based on a pyrolysis process. The analytical boat containing the sample was placed into the instrument, and automatic combustion was performed. The samples were burned at a temperature of 750 °C in an oxygen atmosphere. This process caused the release of mercury (Hg) vapor, which a gold amalgamator captured. The released mercury was measured under the following conditions: a drying time of 150 s, a decomposition time of 150 s, and a waiting time of 45 s. The quantitative determination of mercury (Hg) was conducted at a wavelength of 253.7 nm. The detection limit (LOD) for mercury was 0.0011 mg·kg^−1^ dry weight (DW), and the limit of quantification (LOQ) was 0.0031 mg·kg^−1^ DW. The analytical procedures were verified by determining the Hg concentrations in reference material samples—ERM-CE 278k mussel tissue (IRMM, Geel, Belgium), and blanks were also included in each sample set. The mean Hg concentration obtained was 101.4% of the reference values. After about 30 samples, a new boat was used after being heated twice to remove potential traces of mercury. The apparatus was calibrated using a polarographic mercury standard in 2% HNO_3_ (Merck, Darmstadt, Germany). The mercury concentrations in the studied mushrooms were reported in mg·kg^−1^ of dry weight (DW). Each measurement was replicated three times, with the results expressed as the arithmetic mean of these three measurements.

The statistical analysis of the results, including the relationships within individual groups, was conducted using Statistica 13.3 software (TIBCO Inc., StatSoft, Kraków, Poland). Data distribution was assessed using the Shapiro–Wilk W test. Since the data were not normally distributed, a non-parametric analysis was performed. The Mann–Whitney U test was employed to compare the differences between groups at the *p* ≤ 0.05 and *p* ≤ 0.01 significance levels. Furthermore, the differences between groups were analyzed using Spearman’s correlation coefficient at the same significance levels.

## 3. Results

In both species examined, higher Hg mean concentrations were recorded in the caps than in the stipes, with 0.048 in *Aureoboletus projectellus*, and 0.055 mg·kg^−1^ DW in *Imleria badia*. Regarding the stipes, their Hg contents were 0.032 and 0.025 mg·kg^−1^ DM, respectively. The statistical data for both species are shown in Table 2.

There were also measured correlations between the size of the specific morphological part of the mushroom studied and Hg concentrations. The results are shown in Table 3 and Table 4.

Although in *Aureoboletus projectellus* there was no correlation between the size of its morphological parts, in *Imleria badia* caps there was a strong positive correlation between both parameters, clearly indicating that the bigger the size of the cap, the more Hg it accumulated. In the stipe of this species, there was no significant dependence between its size and Hg concentration.

## 4. Discussion

Harvesting the forest undergrowth, including mushrooms, for personal use in Poland is permitted in forests and not subject to restricted access. Individuals engage in this activity at their own risk. There are restrictions on mushroom picking based on the species, particularly those under strict or partial protection, as outlined in the Regulation of the Minister of the Environment dated 9 October 2014 on the protection of mushroom species [51].

According to data published by the Polish Chief Sanitary Inspectorate [4], the statistically average Pole eats a few kilograms of forest mushrooms per year (commercially available and self-harvested). However, in some areas of Poland, this consumption can reach up to 35 kg per year [52]. In this context, estimating the toxicological risks associated with their consumption seems reasonable, as dangerous elements, including toxic metallic elements such as Hg, are found in mushrooms [36,37]. Every year, thousands of tons of mushrooms are sold in various forms, including dried, pickled, fried, and frozen varieties. In Poland, mushroom sales surge during the season. In 2018, buyers purchased 3261 tons of mushrooms, with 555 tons being from the Pomeranian Voivodeship alone. Among the different species available, the brown mushroom ranked highest, accounting for 2057 tons of the total purchases, which included 360 tons from the Pomeranian Voivodeship [53].

Ronda et al. [52] found that the mean mercury content of *Imleria badia* mushrooms ranged from 0 to 0.1 mg·kg^−1^ DW and was the lowest among the other edible mushroom species, such as *Cantharellus cibarius*, *Xerocomus subtomentosus*, and *Suillus luteus*, tested by the authors in Poland. The mercury content recorded in the mushroom samples in the present study is similar, as it falls within this range. Gąsecka et al. [54] showed that the concentration of elements in the soil reflects their content in the fruiting bodies of *Imleria badia*. They also found significantly higher mean Hg concentrations in the fruiting bodies of fungi from heavily contaminated areas, such as the Silesian Region (1.18 mg·kg^−1^ DW), than those from unpolluted ones, such as Wielkopolska National Park in Poland (0.19 mg·kg^−1^ DW).

A comparison of our results with those of other authors shows that the *Imleria Badia* basidiomes analyzed have a low Hg concentration, indicating that the area from which they were collected was unpolluted. The mean Hg content of *Aureoboletus projectellus* is also low and indicates an uncontaminated area, but there are no data in the literature for this species. Furthermore, the mean Hg contents of the two fungal species are not practically different. Gąsecka et al. [54] concluded that *Imleria badia* should not be collected for consumption from any areas that may be contaminated: urban, industrial, or in their vicinity. This fact is also emphasized by Demková et al. [46]. Mleczek et al. [37] assessed the content of Hg in *Imleria badia* from Polish forests from 1974 to 2019. They showed an average content of 0.323 mg·kg^−1^ DW that remained at a similar level throughout the entire study period of 45 years. The range of data varied from 0.064 to 0.824 mg·kg^−1^ DW, and these values were significantly the lowest among the other fungi studied: *Boletus edulis*, *Leccinum scabrum*, and *Macrolepiota procera*. In our study, the mercury concentrations of fungal fruiting bodies ranged from 0.004 to 0.179 and from 0.001 to 0.2 mg·kg^−1^ DW for *Imleria badia* and *Aureoboletus projectellus*, respectively. These values were lower than those reported by Mleczek et al. [37]. However, these authors emphasized that all of the fungal species studied by them were characterized by high BCF values (>1 indicates the accumulation of the element) for Hg. The significant difference (*p* ≤ 0.01) between the mean Hg content in the stipes and caps of both species studied is noteworthy. It turns out that the cap contained a higher Hg content than the stipe, in the case of *Aureoboletus projectellus*, by more than two times, and in the case of *Imleria badia*, by 2.8 times, when comparing the median Hg concentrations. The same relationship was shown by Demková et al. [46] in the fruiting bodies of *Imleria badia*, in which higher median values for Hg were found in the caps (1.09) than in the stipes (0.49), and this was confirmed by the Spearman correlation coefficient results indicating a strong correlation (0.78) between the parts of the mushroom evaluated. At the same time, the authors showed that of the three species studied in Slovakia, *Imleria badia* had the highest Hg content compared with *Boletus subtomentosus* and *Xerocomellus chrysenteron*. The oral reference dose (RfD) for mercury that a person can take throughout their lifetime without any risk of adverse health effects is set at 0.0003 mg·kg^−1^ per body weight daily [46]. The reference dose estimates the daily intake of a single substance that does not pose a risk, despite being consumed over a lifetime. Thus, for an average person (weighing 70 kg), the maximum weekly mercury intake is 0.0003 × 70 kg × 7 days, or 0.147 mg. In line with JECFA, the CONTAM Panel established a tolerable weekly intake (TWI) for inorganic mercury of 4 µg·kg^−1^ b.w. (0.004 mg) [44]. Calculated on a body weight of 70 kg, this gives 0.28 mg inorganic mercury. Given the highest value recorded and the degree of hydration of the mushrooms, achieving the TWI value would require eating approximately 2.8 kg of mushrooms weekly, which is a very high value. If the average values are taken into account, the consumption of the tested mushrooms does not carry a toxicological risk. In this context, it is worth comparing our results with the data on the maximum mercury residue levels in other foods published in Regulation (EC) No. 396/2005 [45]. On 18 January 2018, new maximum residue levels were published in the Official Journal of the European Union in Regulation (EU) No. 2018/73, valid from 7th February 2018 [55]. The quoted act states that recent studies have shown that residues of mercury compounds occur in cultivated fungi at 0.05 mg·kg^−1^ fresh mass and in wild fungi at 0.50 mg·kg^−1^. Therefore, the values recorded in our study can be considered low and comparable with the values for cultivated mushrooms. To support the thesis that the mushrooms studied do not pose a health risk regarding Hg concentration, the target hazard quotient (THQ) for the highest mean recorded value found in the caps of *Imleria badia* was calculated. THQ = (Efr × ED × ADC × CE/RfDo × BW × ATn)/10^−3^, where Efr is the frequency of exposure (365 days); ED is the exposure duration estimated at 74 years; ADC is the average daily consumption of mushrooms, which, according to Central Statistical Office in Poland, is 27 g/day; CE is the average Hg concentration in mushroom samples (mg·kg^−1^); and RfDo is the oral reference dose for mercury (0.0003 mg·kg^−1^ day^−1^). BW is the average body weight (70 kg), ATn covers the average exposure time (365 days × 74 years, men’s life expectancy in Poland = 27,010 days), and 10^−3^ is a factor of the unit’s conversion. However, it should be considered that the estimated value is an approximation, as many of the factors that make up the final result are variable. First of all, mushroom consumption is a variable value, being a result of cultural considerations, place of residence, and many other factors, such as the availability of mushrooms due to seasonality and climatic conditions in particular years, as well as life expectancy, which in Poland, for women, is 82 years and for men 74 years. Nevertheless, it is generally accepted that carcinogenic health effects are not expected if the THQ is lower than 1. If the THQ is more significant than 1, there is a risk of revealing toxic effects, including carcinogenicity. Considering the above calculations, the THQ resulting from the consumption of mushrooms harvested on the central coast of Poland is 0.07.

The mercury content of the mushroom samples varied slightly between species and was mainly at low levels. However, in the tissues of animals that consume mushrooms, for example, wild ruminants such as roe deer, red deer, and wild boar, Hg levels can increase and consequently threaten game consumers due to biomagnification in the trophic chain [56]. Nevertheless, comparing our results to the other investigations, it can be stated that forests of the central Polish coast are areas not polluted with mercury [31,36,46]. This is mainly due to the low human population density and low industrialization. Tourists periodically populate the area in question, but their stay in the area is limited for climatic reasons, mostly to two months a year [57], and mushrooms harvested en masse at this time can be considered a healthy product with no toxicological exposure to elevated mercury concentrations.

## 5. Conclusions

The present study was carried out to investigate the accumulation of total mercury in two species of edible forest mushrooms on the Polish central coast. The values recorded were low when compared with the results of other researchers. Nevertheless, there were statistically significant differences in Hg concentrations between the morphological parts of mushroom fruiting bodies. In both species, caps had higher Hg concentrations than stipes. None of the analyzed species pose a health risk from the perspective of TWI. The consumption of mushrooms picked at the Polish shore can be considered safe regarding health risks caused by Hg, even during long-term consumption. The research conducted drives us to the conclusion that forest mushrooms representing the investigated species can be a valuable addition to the human diet, while at the same time are a natural product that is desirable not only for its taste and health-promoting properties, but also for its lack of a negative impact on the environment. In addition, this is the first attempt to assess the Hg concentrations of the fruiting bodies of a new species inhabiting Central Europe’s forests, *Aureoboletus projectellus*, and to compare it with native species.

## Figures and Tables

**Table 1:** Characteristics of harvested mushrooms (measurements expressed in mm).

Parameter	*Aureoboloetus projectellus* *n* = 102		*Imleria badia* *n* = 92	
	Cap diameter	Stipe length	Cap diameter	Stipe length
Average	46.77	79.48	61.17	52.89
Median	42.50	73.50	50.00	48.50
Minimum	15.00	40.00	17.00	30.00
Maximum	145.00	170.00	115.00	110.00
Q_25_	30.00	62.00	29.00	39.00
Q_75_	61.00	90.00	100.00	65.00
SD	21.80	28.82	34.65	18.07

Q_25_—lower quartile, Q_75_—upper quartile, SD—standard deviation.

**Table 2:** The mercury concentrations in the two mushroom species studied (mg·kg^−1^ DW).

Species	Part	Statistical Parameters							
		*n*	Average	Median	Min	Max	Q_25_	Q_75_	SD
*Aureoboletus projectellus*	Cap	102	0.048 A	0.043	0.001	0.116	0.025	0.066	0.026
	Stipe		0.032 B	0.019	0.005	0.200	0.015	0.033	0.033
*Imleria badia*	Cap	98	0.055 A	0.043	0.004	0.179	0.028	0.084	0.037
	Stipe		0.025 B	0.015	0.006	0.087	0.009	0.040	0.022

A, B—statistically significant differences at *p* ≤ 0.01.

**Table 3:** Correlation between size and Hg concentration in both morphological parts of American boletus.

*Aureoboletus projectellus*	Stipe	Cap
Size	−0.178	0.196

**Table 4:** Correlation between size and Hg concentration in both morphological parts of brown boletus.

*Imleria badia*	Stipe	Cap
Size	0.018	0.613 *

*—statistically significant difference at *p* ≤ 0.01

## Data Availability

The original contributions presented in the study are included in the article. Further inquiries can be directed to the corresponding authors.
